# Orthogonality Measurement of Three-Axis Motion Trajectories for Micromanipulation Robot Systems

**DOI:** 10.3390/mi12030344

**Published:** 2021-03-23

**Authors:** Yuezong Wang, Jinghui Liu, Hao Chen, Jiqiang Chen, Yangyang Lu

**Affiliations:** 1 Faculty of Materials and Manufacturing, Beijing University of Technology, Beijing 100124, China; S201801190@emails.bjut.edu.cn (H.C.); chenjiqiang@emails.bjut.edu.cn (J.C.); luyangyang@emails.bjut.edu.cn (Y.L.); 2 School of Emergency Management, Institute of Disaster Prevention, Sanhe 065201, China

**Keywords:** stereo light microscope, microscopic stereovision, micromanipulation, motion orthogonality

## Abstract

In robotic micromanipulation systems, the orthogonality of the three-axis motion trajectories of the motion control systems influences the accuracy of micromanipulation. A method of measuring and evaluating the orthogonality of three-axis motion trajectories is proposed in this paper. Firstly, a system for three-axis motion trajectory measurement is developed and an orthogonal reference coordinate system is designed. The influence of the assembly error of laser displacement sensors on the reference coordinate system is analyzed using simulation. An approach to estimating the orthogonality of three-axis motion trajectories and to compensating for its error is presented using spatial line fitting and vector operation. The simulation results show that when the assembly angle of the laser displacement sensors is limited within a range of 10°, the relative angle deviation of the coordinate axes of the reference coordinate frame is approximately 0.09%. The experiment results show that precision of spatial line fitting is approximately 0.02 mm and relative error of the orthogonality measurement is approximately 0.3%.

## 1. Introduction

Stereo light microscopes (SLMs) consist of two independent optical paths that form a binocular microscopic stereovision system. This kind of stereovision system not only possesses a long working distance, but can also capture dynamic-scene images in real time. Therefore, it is suitable for microscopic fields, and it can be combined with motion control systems and micromanipulators to form automatic micromanipulation robot systems, which are employed to capture, transfer, or assemble some microscale objects (such as biological tissues, cells, microstructures, etc.). Their sizes range from microns to submillimeter, and the positioning precision of the system can reach micron or even submicron or nanometer levels. This kind of system has been applied in microassembly [1,2,3], microinjection [4,5], bioengineering [6,7,8,9,10,11], minimally invasive surgery [12,13,14,15], and other microscopic fields. The motion control system with three translational degrees of freedom and driven by ball screw, guide rail, slider, and motor, is one of the various motion control systems. It has a simple structure, can provide small displacement in three orthogonal directions, and can drive the micromanipulator to move in a small length range. Its control program design is flexible, and the displacement can be adjusted under various resolutions. This kind of motion control system is convenient to use in micromanipulation robot systems [16,17]. It contains three motion axes, which are equivalent to the three axes in the Cartesian coordinate system. Theoretically, when moving along these three axes or in the same direction, their trajectories should be straight lines and orthogonal to each other. The better the linearity and orthogonality of the motion trajectory lines on the motion axes, the higher the positioning precision. However, due to the machining accuracy of the ball screw and other transmission errors, the real motion control system cannot achieve absolute orthogonality and straightness of the motion trajectory. Therefore, these two factors need to be measured and corrected in high-precision micromanipulation robot systems.

The basic unit of a motion control system is the linear motion module. One-dimensional linear motion modules are composed of a ball screw, guide rail, and motor. According to the orthogonal assembly relationship, three motion modules are combined to form a three-axis motion control system. The positioning precision of this motion control system is inevitably affected by the machining accuracy of main parts such as ball screw and guide rail. Considerable research has been conducted on the error source, error distribution, error modeling, error analysis, and error compensation of the ball screw and guide rail [18,19,20,21,22]. Before leaving the factory, the manufacturer usually conducts orthogonal experiments on the linear motion module assembly using auxiliary tools such angle rulers and angle measuring instruments to measure the installation angles between the linear motion modules to ensure the orthogonal relationship of installation angles much far as possible. However, in the motion process, the three-axis linear motion module group is affected by a variety of error sources (such as the machining precision of ball screws and guide rails; the transmission error between ball screws, guide rails, and sliders; the assembly’s orthogonal error; etc.), so its trajectories on three motion axes are not strictly in a straight line and orthogonal. Especially when these linear motion modules are integrated into the micromanipulation robot system, they need to be reassembled, which will break the original assembly relationship. Therefore, in micromanipulation robot systems with high precision positioning requirements, the trajectories of the motion axes are measured by obtaining the position coordinates of points on the motion trajectories, and the orthogonality and straightness of the trajectories of the motion axes are analyzed to provide data for the calibration of the orthogonality and straightness of the motion axis, which are helpful for improving the positioning precision of motion control systems.

Laser interferometry methods can be used for length measurement in precision environments [23,24]. They use the principle of light interference to generate interference fringes. By analyzing the patterns of interference fringes to measure the distance, high measurement accuracy can be achieved (nanometer and micron levels, etc.). For example, these methods are used to measure and compensate for the geometric error and nonlinear force error of high-speed, high-precision, and large-scale motion control system of mounters [25]. When using laser interferometry to measure the error of the motion control system, the measurement and compensation accuracy can reach the micron level. Laser interferometry methods can also be used for position tracking and measurement of three-axis motion control systems with a ball screw and motor, but the cost of the interference measurement equipment is high and the equipment requires sufficient installation space. The binocular stereo vision method can also be used to track spatial position and measure trajectory [26,27]. A calibration object is mounted on the three-axis motion control system to make the calibration object move along the three motion axes. The stereo image pairs of the calibration object are obtained by a binocular camera. The motion trajectories of the calibration object can be reconstructed based on stereo matching and visual calculation. This kind of measurement system is easy to build, low cost, and easy to use, but the measurement accuracy depends on the accuracy of image processing and stereo vision calculation. In recent years, laser displacement sensors based on the principle of triangulation have developed rapidly, with the emergence of point sensors, stripe-structured light sensors, etc., which can detect the position of a single point or multiple simultaneously and are being increasingly widely used for many tasks such as shape reconstruction, geometric dimension measurement, distance measurement, etc. [28,29,30,31]. The design of the laser displacement sensor is modularized and compact. It can provide displacement data of different ranges and precisions; it is also cheap and a compact position tracking and measurement system is easy to build. The working space of the micromanipulation robot system is narrow and small, and the system for measuring the trajectory of the motion axis is required to have a small and compact structure, so laser displacement sensors are suitable for this demand.

Focusing on the three-axis motion control system used in micromanipulation robot systems, the purpose of this study was to construct a method to measure and analyze the orthogonality and linearity of the trajectories of the motion axes. Based on a laser displacement sensor with a small spot, a spatial position tracking system was designed to measure the trajectories of the motion axes. On this basis, an evaluation method of the orthogonality and linearity of the trajectory of the motion axis was established. The method is low-cost, easy to use, and suitable for micromanipulation. In addition, it can be used as an on-site measurement system without too much calibration. Because laser displacement sensors are general module that have a variety of ranges and precision, the method can also meet different range and precision requirements (such as submicron, micron, tens of microns, etc.), which is very flexible.

The remainder of this article is organized as follows: In Section 2, the micromanipulation robot system is introduced; in Section 3, the method for three-axis orthogonality measurement is developed. In Section 4, the orthogonal reference coordinate system is analyzed. This method is discussed in Section 5, and the conclusions are described in Section 6.

## 2. Motion Trajectory Coordinate Frame

**Figure 1:**
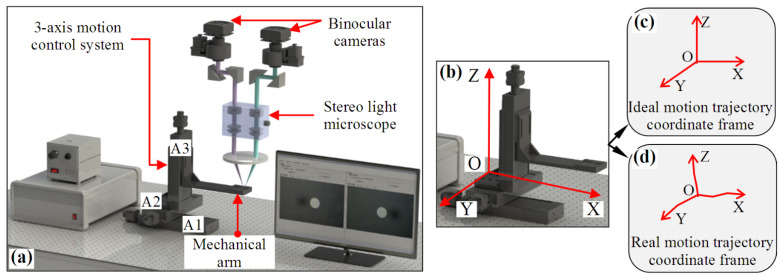
The setup of one kind of micro-manipulation system using a 3-axis motion control system. (**a**) General composition of the system, A1: X-axis linear motion module, A2: Y-axis linear motion module, A3: Z-axis linear motion module. (**b**) Definition of the motion trajectory coordinate frame. (**c**) For an ideal motion trajectory coordinate frame, when moving along its three motion axes (X-axis, Y-axis and Z-axis), the trajectories should be straight and orthogonal. (**d**) For a real motion trajectory coordinate frame, when moving along its three motion axes, the trajectories may be curves with slight deformation, and the perpendicularity of the fitting trajectory lines may deviate by 90 degrees.

Figure 1a shows a special robotic micromanipulation system [16,17,26] that uses a stereo light microscope and binocular cameras to form a microscopic binocular stereovision system, which captures stereo image pairs of space scene in real time, and realizes three-dimensional positioning, navigation, position feedback, etc. In this system, the three-axis motion control system is used as the positioning driver, which receives the position coordinates reconstructed by the binocular stereovision system to achieve accurate positioning. Various types of micromanipulators can be carried on its mechanical arm to perform tasks such as microgripping, microassembly, microinjection, etc. The three-axis motion control system is used not only as the coordinate acquisition device of control points for system calibration, but also as the driving device of position adjustment.

The three-axis motion control system in Figure 1a is composed of three linear motion modules, which are assembled according to the orthogonal relationship. Each linear motion module is mainly composed of a ball screw, double linear guide, coupling, and a motor. To describe the position coordinates of the three-axis motion control system in the motion process, the motion trajectory coordinate frame is defined as shown in Figure 1b. The motion trajectory coordinate frame is represented by O-XYZ, where O is the origin, and X, Y, and Z represent the three motion coordinate axes. At the starting position of each linear motion module, a triggered switch sensor is installed to generate an absolute zero point (or the zero point of grating ruler installed on linear motion module is used as the zero point of the module), and the position of the absolute zero points of the three motion modules is used as the origin of the coordinate frame, i.e., point O. The trajectories of the three motion axes are generated as follows: At one time, only one linear motion module is allowed to move in a line, and the other two linear motion modules remain stationary. The trajectory of the moving module forms a motion coordinate axis. According to this rule, modules A1, A2, and A3 move in a line separately, and their trajectories form the three motion coordinate axes: X-, Y-, and Z-axes. If none of the error factors interfere with the motion, the trajectory lines of the X-, Y-, and Z-axes should be lines; after the three linear motion modules are assembled according to the orthogonal relationship, the trajectory lines of the three motion axes are orthogonal, so the motion coordinate frame at this time is a typical Cartesian coordinate frame, as shown in Figure 1c. However, the motion control system is actually affected by a variety of error factors in the motion process such as: (1) when the vertical installation angles between the three linear motion modules deviate 90°, the motion trajectory lines on the X-, Y-, and Z-axes cannot guarantee perpendicularity. In the assembly process, this error factor will only be reduced as much as possible, but will not disappear. (2) The machining precision of the ball screw and guide rail affects the straightness of the motion trajectory on the *X*-axis, the *Y*-axis and the *Z*-axis, which may cause small deformation of the motion trajectory line. This error source also affects the orthogonality of the motion trajectory line. (3) The assembly error among the ball screw, guide rail, and slider also affects the linearity and orthogonality of motion trajectory line on the motion coordinate axis. Only a few error sources are introduced above, but their effects do exist. Due to the influence of error factors, the motion trajectory coordinate frame is no longer a Cartesian coordinate frame in terms of orthogonality and coordinate axis linearity, as shown in Figure 1d. The posture of deviation from orthogonality and line is relatively small. It is not necessary to analyze and correct the orthogonality and coordinate axis linearity for all three-axis motion control systems, only when there is a need for high-precision positioning is this analysis is more necessary, such as the system in Figure 1a.

The motion of the three-axis motion control system is realized by a control algorithm, which is designed based on the Cartesian coordinate frame in Figure 1c. It can be imagined that when the three-axis motion control system moves in the coordinate frame in Figure 1d, it cannot accurately reach the preset position by the control algorithm, so for the system in Figure 1a, it is necessary to analyze the orthogonality and linearity of the motion trajectory coordinates. Therefore, we developed an orthogonality and linearity evaluation method.

## 3. Methods

**Figure 2:**
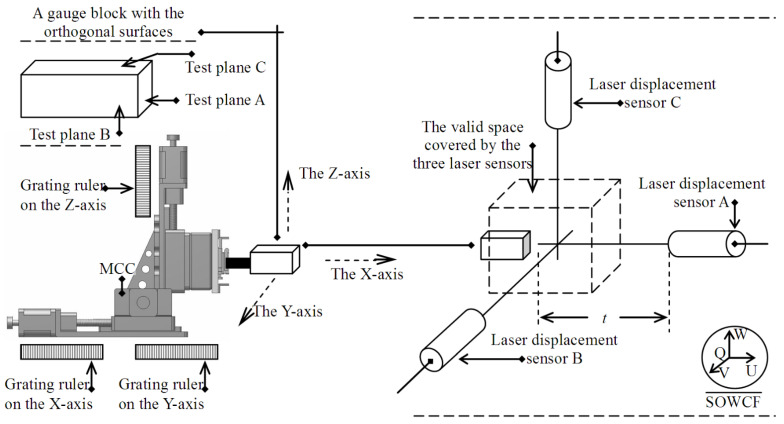
The setup to estimate the intersection angles of the coordinate axes of the motion trajectory coordinate frame, where SOWCF = standard orthogonal world coordinate frame.

A schematic diagram of three-axis motion trajectory measurement is shown in Figure 2. Three grating-ruler sensors with a resolution of 0.1 μm were precisely installed on the X, Y, and Z linear motion modules of the motion control system to measure small displacement in each motion axis. The origin of the motion trajectory coordinate frame O-XYZ was set on the motion control system, and the X-, Y-, and Z-axes coincided with the motion trajectories of the three linear motion modules. The motion trajectories can be regarded as a series of spatial discrete points, whose spatial coordinates are obtained by the measurement system, as shown in Figure 2. A cuboid gauge block composed of stainless steel was installed on the motion control system, and its three adjacent surfaces, *A*, *B*, and *C*, were used as the test surfaces for displacement measurement. These test surfaces had good surface roughness and maintained accurate orthogonality. 

When assembling the gauge block, the normal direction of its three test surfaces should be parallel to the X-, Y-, and Z-axes as much as possible. That is, the normal directions of the three test surfaces *A*, *B*, and *C* should be parallel to the trajectory lines of the three motion axes of the motion control system as much as possible. The gauge block followed the motion control system; moved along the X-, Y-, and Z-axes at intervals; and stayed at the preset location for a certain time. Then, three laser displacement sensors (HG-C1050, Panasonic, Tokyo, Japan) were used to measure the displacement of the three test surfaces simultaneously. Three laser displacement sensors (A, B, and C) were used to construct the motion trajectory measurement system to measure the displacement of the three test surfaces (A, B, and C). During assembly of the three laser displacement sensors, the central-axis lines of the sensors should satisfy the orthogonal condition as much as possible. The non-orthogonality of laser sensor assembly influences the displacement measurement and is discussed in Section 4. The three laser displacement sensors constitute an approximate orthogonal world coordinate frame denoted by Q-UVW, where Q is the origin; U, V, and W represent the three axes; and the measured motion trajectories of the gauge block are described in Q-UVW. The spatial trajectory lines corresponding to the motion trajectories were obtained by the spatial linear fitting method, and the vector parameters of the spatial lines were derived from spatial line equations. Then, the angles between these spatial trajectory lines were calculated by vector calculation and the orthogonality of the three coordinate axes of O-XYZ were estimated. If the coordinate axes of O-XYZ do not satisfy the orthogonal conditions, it becomes a non-orthogonal coordinate frame and its coordinates should be corrected based on the orthogonality measurement. These coordinates in the real non-orthogonal O-XYZ are transformed into a virtual orthogonal coordinate system; then, accurate positioning control is achieved. The valid detection ranges of the three laser displacement sensors are depicted by dotted lines in Figure 2. The gauge block only moves in the valid space.

### 3.1. Data Acquisition

**Figure 3:**
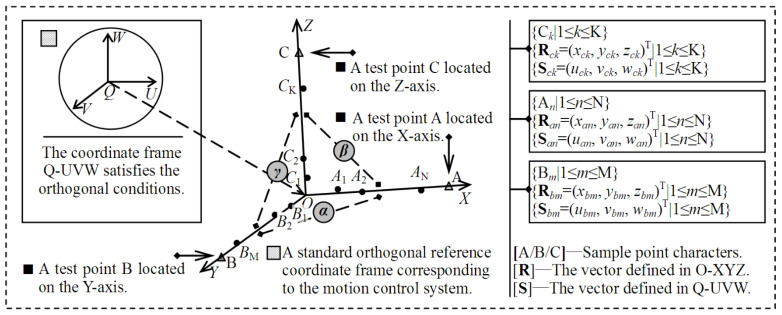
The method to estimate the intersection angles between the coordinate axes of the motion trajectory coordinate frame.

The three laser displacement sensors were used to construct the approximate orthogonal world coordinate frame Q-UVW, as shown in Figure 2. To measure the trajectories on the motion axes, the gauge block moved along the preset trajectories in the valid cuboid space, and the displacement of the test surface was measured. In Figure 2, **S** represents the vector of a point on the test surface in Q-UVW, and is generated by the laser displacement sensors; **R** represents its vector in O-XYZ, and is generated by the three grating-ruler sensors. The motion trajectory is composed of a series of separate points, and the coordinate vectors of all the separate points constitute a data sample, which is the basic data of the orthogonality estimation of the three-axis motion trajectories of O-XYZ. Figure 3 shows the acquisition of data samples.

The gauge block moved at preset distance intervals on the X-, Y-, and Z-axes, or in directions parallel to them. In Figure 2, {A*_n_*|1 ≤ *n* ≤ N} represents the set consisting of *N* points on the *X*-axis trajectory and A*_n_* is an element. Point A*_n_* corresponds to two vectors: one is defined in O-XYZ, and the other is defined in Q-UVW. So, the set {A*_n_*|1 ≤ *n* ≤ N} corresponds to two coordinate vector samples: {**R***_an_* = (*x_an_*, *y_an_*, *z_an_*)^T^|1 ≤ *n* ≤ N} and {**S***_an_* = (*u_an_*, *v_an_*, *w_an_*)^T^|1 ≤ *n* ≤ N}. The vector **S***_an_* consists of three elements, *u_an_*, *v_an_*, and *w_an_*, whose values are the three displacement components of the gauge block at point A*_n_*. **S***_an_* is measured by the three laser displacement sensors. Similarly, {B*_m_*|1 ≤ *m* ≤ M} and {C*_k_*|1 ≤ *k* ≤ K} are the point sets corresponding to the Y- and *Z*-axis trajectories, respectively, and their vector sets are {**R***_bm_* = (*x_bm_*, *y_bm_*, *z_bm_*)^T^|1 ≤ *m* ≤ M} and {**S***_bm_* = (*u_bm_*, *v_bm_*, *w_bm_*)^T^|1 ≤ *m* ≤ M}, respectively.

### 3.2. Three-Axis Orthogonality Evaluation

In this section, a spatial linear fitting approach is applied to the vector sets {**S***_an_*|1 ≤ *n* ≤ N}, {**S***_bm_*|1 ≤ *m* ≤ M}, and {**S***_ck_*|1 ≤ *k* ≤ K}, which outputs the line equations and their parameters. The intersection angles between the fitted lines are calculated based on vector operation, and the orthogonality of the fitted lines in O-XYZ is analyzed based on these angles. The intersection angles between the X- and *Y*-axis, between the X- and *Z*-axis, and between the Y- and *Z*-axis are denoted by *α*, *β*, and *γ*, respectively, as shown in Figure 3, the values of which indicate the orthogonal levels between the coordinate axes. The classification criteria related to the orthogonality are proposed and listed in Table 1.

The angles *α*, *β*, and *γ* are compared with 90°. The orthogonality condition is satisfied if the differences between these angles are less than the threshold *T_abc_*; instead, the condition cannot be satisfied.

**Figure 4:**
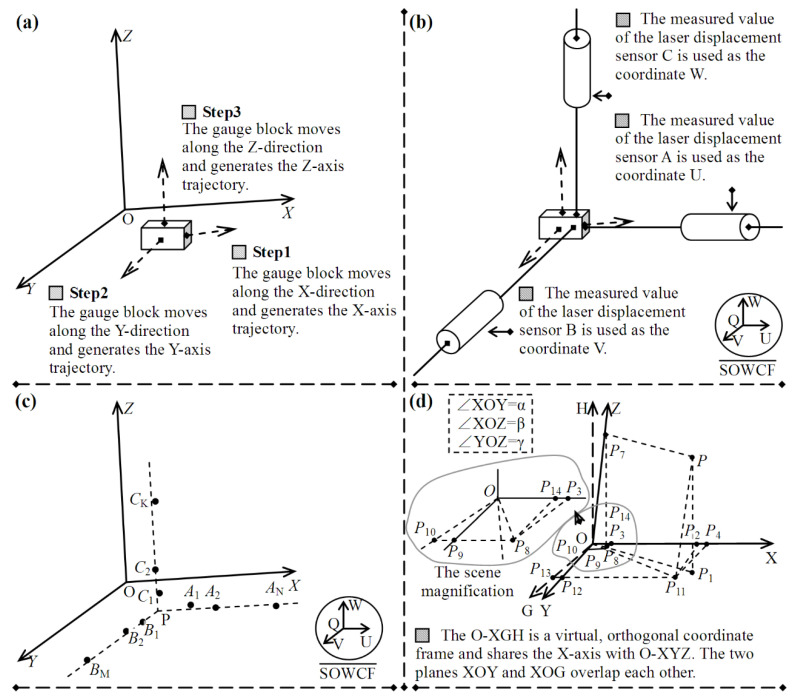
The procedure of the orthogonality estimation and the orthogonality correction. (**a**) The motion control system carries the gauge block to move along the X-direction, the Y-direction and the Z-direction. (**b**) The variations of the displacement are measured by the three laser displacement sensors. (**c**) The test points are used to simulate the motion trajectories, and the intersection angles of the motion trajectories are calculated. (**d**) The intersection angles of the motion trajectories obtained by the steps (**a**–**c**) are used to correct the coordinates of the motion control system.

Figure 4 shows the process of the orthogonality estimation, which includes the following steps:

*Step 1*: The data acquisition process is conducted; the three sets are generated, {A*_n_*|1 ≤ *n* ≤ N}, {B*_m_*|1 ≤ *m* ≤ M}, and {C*_k_*|1 ≤ *k* ≤ K}; and their vector sets are measured, as shown in Figure 4a,b.

*Step 2*: A spatial linear fitting approach is applied to the six vector samples in Figure 3. The linear equations and their parameters are obtained, and the error of spatial line fitting is calculated, as shown in Figure 4c.

*Step 3*: The angles *α*, *β*, and *γ* are calculated based on the parameters, and the orthogonality between the coordinate axes is analyzed on the basis of the principles defined in Table 1.

A general expression of spatial line equation is defined as follows:

(1) $$\frac{u-u_{0}}{\rho_{u}}=\frac{v-v_{0}}{\rho_{v}}=\frac{w-w_{0}}{\rho_{w}}$$

where (*u*, *v*, *w*)^T^ is the coordinate vector of a point located in Q-UVW, (*u*_0_, *v*_0_, *w*_0_)^T^ is the coordinate vector of a known reference point, and (*ρ_u_*, *ρ_v_*, *ρ_w_*)^T^ is the unit vector of the spatial line. The above six parameters describe a spatial line and constitute a parameter vector **P**, where **P** = (*u*_0_, *v*_0_, *w*_0_, *ρ_u_*, *ρ_v_*, *ρ_w_*)^T^. The fitted lines based on the three sets, {**S_an_**|1 ≤ *n* ≤ N}, {**S_bm_**|1 ≤ *m* ≤ M}, and {**S_ck_**|1 ≤ *k*≤ K}, can be described by the following general expression:

(2) $$\{\begin{array}{c} P_{a}=F\left(\left\{S_{an}|1\leq n\leq N\right\}\right)\\ P_{b}=F\left(\left\{S_{bm}|1\leq m\leq N\right\}\right)\\ P_{c}=F\left(\left\{S_{ck}|1\leq k\leq N\right\}\right) \end{array}$$

where *F* represents spatial line fitting method; **P_a_**, **P_b_**, and **P_c_** are the three parameter vectors of **P**, where **P_a_** = (*u*_0,*a*_, *v*_0,*a*_, *w*_0,*a*_, *ρ_u_*_,__*a*_, *ρ_v_*_,__*a*_, *ρ_w_*_,__*a*_)^T^, **P_b_** = (*u*_0,*b*_, *v*_0,*b*_, *w*_0,*b*_, *ρ_u_*_,__*b*_, *ρ_v_*_,__*b*_, *ρ_w_*_,__*b*_)^T^, and **P_c_** = (*u*_0,*c*_, *v*_0,*c*_, *w*_0,*c*_, *ρ_u_*_,__*c*_, *ρ_v_*_,__*c*_, *ρ_w_*_,__*c*_)^T^. Deng et al. [32] proposed a mixed least-square algorithm to fit a spatial line, which was used to fit the sets {**S_an_**|1 ≤ *n* ≤ N}, {**S_bm_**|1 ≤ *m* ≤ M}, and {**S_ck_**|1 ≤ *k* ≤ K} in this study. If the three unit vectors of the fitted lines are represented by **n_a_**, **n_b_**, and **n_c_**, where **n_a_** = (*ρ_u_*_,__*a*_, *ρ_v_*_,__*a*_, *ρ_w_*_,__*a*_)^T^, **n_b_** = (*ρ_u_*_,__*b*_, *ρ_v_*_,__*b*_, *ρ_w_*_,__*b*_)^T,^ and **n_c_** = (*ρ_u_*_,__*c*_, *ρ_v_*_,__*c*_, *ρ_w_*_,__*c*_)^T^, the intersection angles *α*, *β*, and *γ*, can be derived using the dot product operation:

(3) $$\{\begin{array}{c} \cos\alpha =n_{a}\cdot n_{c}\\ \cos\beta =n_{a}\cdot n_{b}\\ \cos\gamma =n_{b}\cdot n_{c} \end{array}$$

### 3.3. Compensation for Three-Axis Nonorthogonality

If the coordinate axes of O-XYZ cannot maintain the approximate orthogonal relationship, a virtual orthogonal coordinate system O-XGH is created, which uses the same *X*-axis as O-XYZ, as shown in Figure 4d. O-XGH is used to compensate for the orthogonal error. Here, plane XOY in O-XYZ and plane XOG in O-XGH overlap. It is assumed that the vectors of a point *P* in O-XGH and O-XYZ are **r**_p,gh_ = (*x*_p,gh_, *y*_p,gh_, *z*_p,gh_)^T^ and **r**_p,yz_ = (*x*_p,yz_, *y*_p,xyz_, *z*_p,yz_)^T^, where the subscripts gh and yz represent O-XGH and O-XYZ, respectively. The vector **r**_p,gh_ is obtained by vision calculation. The vector **r**_p,yz_ represents the real displacement of the motion control system and is given by grating-ruler sensors. The purpose of compensation for the non-orthogonal coordinate frame O-XYZ is to transform **r**_p,gh_ to **r**_p,yz_, and a general expression between them is defined as:

(4) $$r_{p,\mathrm{yz}}=Method(r_{p,\mathrm{gh}})$$

where *Method*() represents the transformation method between **r**_p,gh_ and **r**_p,yz_. To deduce *Method*(), a rotational transformation is applied to the coordinates in O-XYZ and O-XGH. Equation (4) indicates that more reasonable motion trajectories can be obtained through *Method*(). The motion control system with a nonorthogonal motion coordinate frame will be able to realize precise positioning.

If the unit vector of the *Y*-axis in O-XGH is represented by **n**_y,gh_ = (cosα_y,gh_, cosβ_y,gh_, cosγ_y,gh_)^T^, where α_y,gh_, β_y,gh,_ and γ_y,gh_ are the directional angles, α_y,gh_ and β_y,gh_ can be derived from Figure 4d, i.e., α_y,gh_ = ∠XOY = α and β_y,gh_ = 90 − ∠XOY = 90 − α. Because the *Y*-axis is perpendicular to the H-axis, γ_y,gh_ equals 90°. Based on the above analysis, the unit vector **n**_y,gh_ can be written as **n**_y,gh_ = (cosα, sinα, 0)^T^. Similarly, if the unit vector of the *Z*-axis in O-XGH is **n**_z,gh_ = (cosα_z,gh_, cosβ_z,gh_, cosγ_z,gh_)^T^, where α_z,gh_, β_z,gh,_ and γ_z,gh_ are the directional angles of the *Z*-axis and α_z,gh_ satisfies the condition α_z,gh_ = ∠XOZ = β, the unit vector **n**_z,gh_ can be written as **n**_z,gh_ = (cosβ, cosβ_z,gh_, cosγ_z,gh_)^T^. The intersection angle between **n**_y,gh_ and **n**_z,gh_ equals γ, and the dot product between **n**_y,gh_ and **n**_z,gh_ is calculated as:

(5) $$n_{y,\mathrm{gh}}\cdot n_{z,\mathrm{gh}}=\cos\alpha \cdot \cos\beta +\sin\alpha \cdot \cos\beta_{z,\mathrm{gh}}=∠YOZ=\cos\gamma$$

The cosine value of β_z,gh_ is derived from Equation (5), i.e., cosβ_z,gh_ = (cosγ-cosαcosβ)/sinα. Meanwhile, a normalization condition among α_z,gh_, β_z,gh_, and γ_z,gh_ is satisfied, i.e., (cosα_z,gh_)^2^ + (cosβ_z,gh_)^2^ + (cosγ_z,gh_)^2^ = 1, and the cosine value of γ_z,gh_ is obtained. The unit vector **n**_z,gh_ can be calculated via the angles α, β, and γ.

The projection transformation method is applied to deduce *Method*(), as shown in Figure 4d. A line that is parallel to the H-axis and passes point P is drawn, which intersects with plane XOG at point P_1_. Point P_1_ is the perpendicular projection of point P. Similarly, another line that is parallel to the *Z*-axis and passes point P is drawn, which intersects with plane XOY at point P_11_. Point P_11_ is the projection of point P. A line passing point P_11_ and perpendicular to the G-axis is set and intersects with the G-axis at point P_13_. Line segment P_11_P_13_ intersects with the *Y*-axis at point P_12_, and points P_13_ and P_12_ are the two projections of point P_11_. Similarly, line segments that all pass point P_11_ are set: one of them is perpendicular to the *X*-axis and intersects with the *X*-axis at point P_2_ and the other is parallel to the *Y*-axis and intersects with the *X*-axis at point P_4_. Then points P_4_ and P_2_, corresponding to O-XGH and O-XYZ, respectively, are the two projections of point P, all of which are located on the *X*-axis. A line segment that passes point P and is parallel to line segment OP_11_ is set and intersects with the *Z*-axis at point P_7_. Point P_7_ is the projection of point P. A line that passes point P_7_ and is perpendicular to plane XOG is drawn, and intersects with plane XOG at point P_8_. The two points P_10_ and P_9_, which are located on the G-axis and the *Z*-axis, respectively, are the projections of point P_8_. The two projection points P_3_ and P_14_ of point P_8_ corresponding to O-XGH and O-XYZ, respectively, are all located on the *X*-axis. The coordinate vectors of point P_7_ corresponding to O-XGH and O-XYZ are represented by **r**_p7,gh_ = (*x*_p7,gh_, *y*_p7,gh_, *z*_p7,gh_)^T^ and **r**_p7,yz_ = (0, 0, *z*_p7,yz_)^T^, respectively. Because the vertical coordinates of points P_7_ and P are equal to each other (i.e., *z*_p7,gh_ = *z*_p,gh_) and point P_7_ is the projection of point P on the *Z*-axis, an equal relationship between *z*_p7,yz_ and z_p,yz_ is satisfied, i.e., *z*_p7,yz_ = z_p,yz_. The unit vector of the *Z*-axis via O-XGH, **n**_z,gh_, is a known vector, then the vector **r**_p7,gh_ is determined by the following equation:

(6) $$r_{p7,\mathrm{gh}}=z_{p7,\mathrm{yz}}\cdot n_{z,\mathrm{gh}}=z_{p,\mathrm{yz}}\cdot n_{z,\mathrm{gh}}$$

The following condition applied to the vector operation is satisfied:

(7) $$OP_{11}=P_{11}P-r_{p,\mathrm{gh}}$$

Due to the geometric relationship, as shown in Figure 4d, line segment **P_11_P** is parallel to line segment OP_7_ and the conditions, |**P_11_P**| = |OP_7_|, **P_11_P** = **r**_p7,gh_, and **OP_11_** = **r**_p11,gh_, are satisfied in O-XGH. If the vector of **r**_p11,gh_ is **r**_p12,gh_ = (*x*_p12,gh_, *y*_p12,gh_, 0)^T^, Equation (7) can be rewritten as:

(8) $$r_{p11,\mathrm{gh}}=r_{p7,\mathrm{gh}}-r_{p,\mathrm{gh}}$$

It is assumed that the vector of point P_11_ in O-XYZ is denoted as **r**_p11,yz_ = (*x*_p11,yz_, *y*_p11,yz_, 0)^T^ and the mapping relationship between **r**_p11,gh_ and **r**_p11,yz_ can be derived. Point P_13_, which is located on the G-axis, is the projection of point P_11;_ the vector of point P_13_ in O-XGH is denoted as **r**_p13,gh_ = (0, *y*_p11,gh_, 0)^T^. Point P_12_, which is located on the *Y*-axis, is the projection of point P_11_; the two vectors of point P_12_ corresponding to O-XYZ and O-XGH are denoted as **r**_p12,yz_ = (0, *y*_p11,yz_, 0)^T^ and **r**_p12,gh_ = (*x*_p12,gh_, *y*_p12,gh_, 0)^T^, respectively. Due to the shape of the triangle OP_13_P_12_ being a right-angled triangle, the following relationship is satisfied:

(9) $$\{\begin{array}{c} |P_{11}P_{12}|=|P_{11}P_{13}|-|P_{12}P_{13}|=|P_{11}P_{13}|-|OP_{13}|/tg∠P_{13}OP_{12}\\ |OP_{12}|=|OP_{13}|/\cos∠P_{13}OP_{12} \end{array}$$

In Figure 4d, the conditions, |P_11_ P_12_| = *x*_p11,yz_, |P_11_P_13_| = *x*_p11,gh_, |OP_12_| = *y*_p11,yz_, |OP_13_| = *y*_p11,gh_, and ∠P_13_O P_12_ = 90 − ∠XOY = 90 − α are satisfied and taken into Equation (9). The following relationship can be derived:

(10) $$r_{p11,\mathrm{yz}}=\Omega_{\alpha }\cdot r_{p11,\mathrm{gh}}=\left(\begin{array}{ccc} 1 & -tg\alpha & 0\\ 0 & 1/\sin\alpha & 0\\ 0 & 0 & 0 \end{array}\right)\cdot r_{p11,\mathrm{gh}}$$

where **Ω_α_** is a 3 × 3 matrix. The two coordinates of point P, *z*_p,yz_ and *z*_p,gh_, corresponding to O-XYZ and O-XGH, respectively, can be derived from the side-angle relationship of the right triangle P_1_PP_11_. The conditions |PP_1_| = *z*_p,gh_ and |PP_11_| = *z*_p,yz_ are satisfied and the angle ∠P_11_P P_1_ equals the angle γ_z,gh_ which is the intersection angle between the Z- and the H-axis. The following relationship can be derived:

(11) $$z_{p,\mathrm{yz}}=z_{p,\mathrm{gh}}/\cos\gamma_{z,\mathrm{gh}}$$

Finally, *Method*() is derived based on Equations (6), (8), (10) and (11) as follows:

(12) $$r_{p,\mathrm{yz}}=\Omega_{\alpha }\cdot [(z_{p,\mathrm{gh}}/\cos\gamma_{z,\mathrm{gh}})\cdot n_{z,\mathrm{gh}}-r_{p,\mathrm{gh}}]+r_{0,\mathrm{gh}}$$

where **r**_0,gh_ = (0,0,z_p,gh_/cosγ_z,gh_)^T^. Based on Equation (12), the vector **r**_p,yz_ in O-XYZ is derived from the vector **r**_p,gh_ in O-XGH. The motion control system can accurately generate the displacement via the vector **r**_p,yz_ and drives the micromanipulator to move from point O to point P; finally, its precision positioning under orthogonal conditions is achieved.

## 4. Analysis of the Orthogonal Coordinate Frame

In this section, the influence of the rotation angles of the laser displacement sensor and gauge block on three-axis orthogonality estimation is analyzed.

### 4.1. Description of Position and Posture

**Figure 5:**
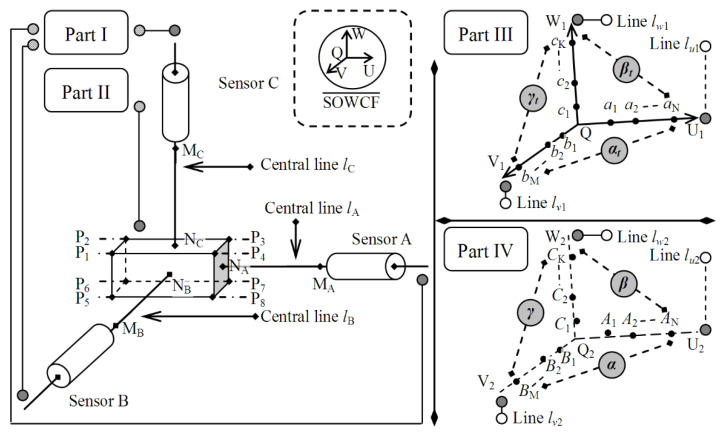
The effects of the poses of the system components on the motion trajectory orthogonality estimation. The error sources include the rotation angles of the sensors in the Part I, and the rotation angles of the standard gauge block in the Part II. The standard gauge block moves at the positions located on the three initial motion trajectory lines in the Part III. And the simulated motion trajectory lines are obtained under the rotations of the sensors and the standard gauge block.

It is assumed that the laser displacement sensors and the gauge block are placed in Q-UVW as shown in Figure 5. In an ideal status, the central-axis lines of the gauge block and the laser displacement sensors and the motion trajectories of the gauge block satisfy the following two constraint rules:

*Constraint rule I*: PART I (Figure 5) shows that the central-axis line of each laser displacement sensor should be perpendicular to the corresponding test surface of the gauge block. For example, the central-axis line *l_A_* of the laser displacement sensor *A* should be perpendicular to test plane Plane_3487_, which is determined by points P_3_, P_4_, P_8,_ and P_7_, where the subscript numbers 3, 4, 8, and 7 of Plane_3487_ represent points P_3_, P_4_, P_8,_ and P_7_, respectively. Similarly, the central-axis lines *l_B_* and *l_C_* of the laser displacement sensors *B* and *C*, respectively, should also be perpendicular to the corresponding test planes Plane_1584_ and Plane_1234_, where plane Plane_1584_ is determined by points P_1_, P_5,_ P_8,_ and P_3_, and plane Plane_1234_ is determined by points P_1,_ P_2,_ P_,_ and P_4_.

*Constraint rule II*: In Q-UVW, the gauge block and its motion trajectories on the coordinate axes should maintain a certain relationship in position and posture. As shown in Part III in Figure 5, the vectors **QU_1_**, **QV_1_**, and **QW_1_** represent the three-axis motion trajectories of the gauge block; and *l_u_*_1_, *l_v_*_1_, and *l_w_*_1_ are their trajectory lines, respectively. During the gauge block moving along line *l_u_*_1_, the vectors of sample points on line *l_u_*_1_ are measured. In this process, test plane Plane_1234_ is parallel to plane U_1_QV_1_, and line P_1_P_4_ or P_2_P_3_ is also parallel to line *l_u_*_1_. During the gauge block moving along line *l_v_*_1_ (or *l_w_*_1_), the coordinates of sample points on line *l_v_*_1_ (or *l_w_*_1_) are measured, and test plane Plane_1234_ (or Plane_1584_) must be parallel to plane U_1_QV_1_ (or U_1_QW_1_), and line P_1_P_2_ or P_3_P_4_ (or P_1_P_5_ or P_7_P_8_) must be parallel to line *l_v_*_1_ (or *l_w_*_1_).

If constraint rules I and II are satisfied simultaneously, the influence of the rotation angles of the laser displacement sensor and gauge block on Q-UVW will decrease. However, it is impossible for the real measurement system to satisfy constraint rules I and II. In other words, it is impossible for lines *l_A_*, *l_B_*, and *l_C_* to absolutely be parallel to the corresponding test planes. Moreover, it is impossible for the central-axis lines of the gauge block to be absolutely parallel to the corresponding trajectory lines. However, the influence of the above error factors on Q-UVW can be minimized as much as possible and limited to a certain range. So, it is necessary to analyze the effects of the position and posture of the gauge block and laser displacement sensors on the measurement.

We define the following parameters for the position and posture of the gauge block and laser displacement sensors. For the gauge block, point P_6_ is set as a point as a basis reference and its coordinate vector in Q-UVW is represented by **s_p6_** = (*u_p_*_6_, *v_p_*_6_, *w_p_*_6_)^T^. Based on point P_6_, the rotation angle vector of the gauge block around the U-, V-, and W-axis is denoted as **Θ** = (*θ_u_*, *θ_v_*,*θ_w_*)^T^. The central-axis lines *l_A_*, *l_B_*, and *l_C_* are rotated by small angles based on different reference points *M_A_*, *M_B_*, and *M_C_*, respectively. The unit vectors of lines *l_A_*, *l_B_*, and *l_C_* are denoted as **n_a_** = (cos*α*_a_, cos*β*_a_, cos*γ*_a_)^T^, **n_b_** = (cos*α*_b_, cos*β*_b_, cos*γ*_b_)^T,^ and **n_c_** = (cos*α*_c_, cos*β*_c_, cos*γ*_c_)^T^, respectively. Lines *l_A_*, *l_B_*, and *l_C_*, respectively, intersect with the corresponding test planes Plane_3487_, Plane_1584_, and Plane_1234_ at points *N_A_*, *N_B,_* and *N_C_*. The vectors of points *M_A_*, *M_B_*, *M_C_*, *N_A_*, *N_B_*, and *N_C_* are denoted as **s_ma_** = (*u_ma_*, *v_ma_*, *w_ma_*)^T^, **s_mb_** = (*u_mb_*, *v_mb_*, *w_mb_*)^T^, **s_mc_** = (*u_mc_*, *v_mc_*, *w_mc_*)^T^, **s_na_** = (*u_na_*, *v_na_*, *w_na_*)^T^, **s_nb_** = (*u_nb_*, *v_nb_*, *w_nb_*)^T,^ and **s_nc_** = (*u_nc_*, *v_nc_*, *w_nc_*)^T^. Based on the above definitions, five vectors, **s_p6_**, **Θ**, **n_a_**, **n_b_** and **n_c_**, are used to describe the position and posture of the gauge block and laser displacement sensors.

The unit vectors of lines *l_u_*_1_, *l_v_*_1_, and *l_w_*_1_ are denoted as **n_u1_** = (cos*α_u_*_1_, cos*β_u_*_1_,cos*γ_u_*_1_)^T^, **n_v1_** = (cos*α_v_*_1_, cos*β_v_*_1_,cos*γ_v_*_1_)^T^, and **n_w1_** = (cos*α_w_*_1_, cos*β_w_*_1_,cos*γ_w_*_1_)^T^, respectively. The intersection angles of lines *l_u_*_1_, *l_v_*_1_, and *l_w_*_1_ are denoted as *α_t_*, *β_t_*, and *γ_t_*, respectively, where *α_t_* = *l_u_*_1_∨*l_v_*_1_, *β_t_* = *l_u_*_1_∨*l_w_*_1_, and *γ_t_* = *l_w_*_1_∨*l_v_*_1_. An angle vector **Λ_t_** = (*α_t_*, *β_t_*, *γ_t_*)^T^ is defined and can be derived by the dot product operation, i.e., *α_t_* = arcos(**n_u1_**·**n_v1_**), *β_t_* = arcos(**n_u1_**·**n_w1_**), and γ_t_ = arcos(**n_w1_**·**n_v1_**). A series of sample points located on lines *l_u_*_1_, *l_v_*_1_, and *l_w_*_1_ are obtained that constitute three sample point sets, which are denoted as {a*_n_*|1 ≤ *n* ≤ *N*}, {b*_m_*|1 ≤ *m* ≤ *M* }, and {b*_k_*|1 ≤ *k* ≤ *K*}, respectively. The corresponding vector sets of the sample point sets are denoted as {**s_a,n_** = (*u_a,n_*, *v_a,n_*, *w_a,n_*)^T^|1 ≤ *n* ≤ *N*}, {**s_b,m_** = (*u_b,m_*, *v_b,m_*, *w_b,m_*)^T^|1 ≤ *m* ≤ *M* }, and {**s_c,k_** = (*u_c,k_*, *v_c,k_*, *w_c,k_*)^T^ |1 ≤ *k* ≤ *K* }, respectively.

### 4.2. Simulation Method

In this section, a simulation method for three-axis motion trajectory estimation is designed based on the MATLAB software. The simulation process consisted of the following steps:

*Step 1***:** In Figure 5, the coordinates of points P_1_–P_8_ were calculated by presetting the values of **Θ** and **s_p6,0_**. The length, width, and height of the gauge block were used as known parameters and denoted as *L_S_*, *W_S_*, and *H_S_*, respectively. The values of *θ_u_*, *θ_v,_* and *θ_w_* were initialized to zero. Then, the initial values of **s_p1,0_-s_p8,0_** were calculated by the following equation:

(13) $$\left(s_{p1,0},s_{p2,0},s_{p3,0},s_{p4,0},s_{p5,0},s_{p6,0},s_{p7,0},s_{p8,0}\right)-s_{p6,0}=\left(\begin{array}{cccccccc} 0 & 0 & L_{s} & L_{s} & 0 & 0 & L_{s} & L_{s}\\ W_{s} & 0 & 0 & W_{s} & W_{s} & 0 & 0 & W_{s}\\ H_{s} & H_{s} & H_{s} & H_{s} & 0 & 0 & 0 & 0 \end{array}\right)$$

After the gauge block was rotated by **Θ**, the new coordinates of **s_pi_** were obtained through the following rotation transformation:

(14) $$s_{pi}=Rot_{u}(\theta_{u})\cdot Rot_{v}(\theta_{v})\cdot Rot_{w}(\theta_{w})\cdot (s_{pi,0}-s_{p6,0})+s_{p6,0}$$

where *i* is the number of the vertex and *i* ∈ [1,8]; **Rot_u_**(*θ_u_*), **Rot_v_**(*θ_v_*), and **Rot_w_**(*θ_w_*) are rotation matrices corresponding to the U-, V-, and W-axis, respectively.

*Step 2*: **n_a_**, **n_b_**, and **n_c_** were initialized by presetting the values of α_a_, β_a_, γ_a_, α_b_, β_b_,γ_b_, α_c_, β_c_, and γ_c_; **s_ma_**, **s_mb_**, and **s_mc_** were calculated by **s_ma_** = (*L_s_* + *L*_0_, *W_s_*/2, *H_s_*/2)^T^, **s_mb_** = (*L_s_*/2, *W_s_*+ *L*_0_, *H_s_*/2)^T^, and **s_mc_** = (*L_s_*/2, *W_s_*/2, *H_s_*+*L*_0_)^T^, where *L*_0_ is a length adjustment parameter.

*Step 3*: The nine angles, *α_u_*_1_, *β_u_*_1_, *γ_u_*_1_, *α_v_*_1_, *β_v_*_1_, *γ_v_*_1_, *α_w_*_1_, *β_w_*_1_, and *γ_w_*_1,_ were initialized and a series of sample points on these lines were obtained. The sample sets were obtained, i.e., {**s_a,n_** = ***S****len_a_* × *n* × **n_u1_|**1 ≤ *n* ≤ *N*}, {**s_b,m_** = ***S****len_b_* × *m* × **n_v1_**|1 ≤ *m* ≤ *M*}, and {**s_c,k_** = ***S****len_c_* × *k* × **n_w1_**|1 ≤ *k* ≤ *K*}, where ***S****len_a_*, ***S****len_b_*, and ***S****len_c_* are the distance between two neighboring sample points.

*Step 4*: The coordinates of the intersection points between the test planes and their corresponding central-axis lines of the laser displacement sensors were calculated. When the gauge block moved along lines *l_u_*_1_, *l_v_*_1,_ and *l_w_*_1_, point P_6_ passed all of sample points in the sets {a*_n_*|1 ≤ *n* ≤ *N*}, {b*_m_*|1 ≤ *m* ≤ *M*}, and {b*_k_*|1 ≤ *k* ≤ *K*}. Other vertices made similar movements with point P_6_. Test planes Plane_3487_, Plane_1584,_ and Plane_1234_ intersected with lines *l_A_*, *l_B_*, and *l_C_* at points *N_A_*, *N_B,_* and *N_C_*, respectively. The laser displacement sensors *A*, *B*, and *C* monitored the coordinate variation in points *N_A_*, *N_B_*_,_ and *N_C_*, primarily involving the coordinates *u_a_* (the U-axis coordinate of point *N_A_*), *v_b_* (the V-axis coordinate of point *N_B_*), and *w_c_* (the W-axis coordinate of point *N_C_*). Then, the position vector of the gauge block, **s_s_,** was given by *u_a_*, *v_b_*, and *w_c_*, where **s_s_** = (*u_a_*, *v_b_*, *w_a_*)^T^.

Part IV in Figure 5 shows that three types of point sets, {A*_n_*|1 ≤ *n* ≤ *N*}, {B*_m_*|1 ≤ *m* ≤ *M*}, and {C*_k_*|1 ≤ *k* ≤ *K*}, were measured. Their coordinate vectors are denoted as {**s_A,n_** = (*u_A,n_*, *v_A,n_*, *w_A,n_*)^T^|1 ≤ *n* ≤ *N*}, {**s_B,m_** = (*u_B,m_*, *v_B,m_*, *w_B,m_*)^T^|1 ≤ *m* ≤ *M*}, and {**s_C,k_** = (*u_C,k_*, *v_C,k_*, *w_C,k_*)^T^ |1 ≤ *k* ≤ *K*}, respectively. When the gauge block reaches the position of a sample point, the normal vectors of planes Plane_3487_, Plane_1584_, and Plane_1234_ can be obtained by the following equation:

(15) $$\{\begin{array}{c} n_{3478}=(s_{p7}-s_{p8})\times (s_{p4}-s_{p8})/(|s_{p7}-s_{p8}|\cdot |s_{p7}-s_{p8}|)\\ n_{1584}=(s_{p4}-s_{p8})\times (s_{p5}-s_{p8})/(|s_{p4}-s_{p8}|\cdot |s_{p5}-s_{p8}|)\\ n_{1234}=(s_{p4}-s_{p1})\times (s_{p2}-s_{p1})/(|s_{p4}-s_{p1}|\cdot |s_{p2}-s_{p1}|) \end{array}$$

The vector **s_s_** of the gauge block at points *N_A_*, *N_B_*, and *N_C_* can be calculated by the following equation:

(16) $$\{\begin{array}{c} s_{na}=s_{ma}+\left\{\left[(s_{ma}-s_{na})\cdot n_{3478}\right]/\left(n_{3478}\cdot n_{na}\right)\right\}\cdot n_{na}\\ s_{nb}=s_{mb}+\left\{\left[(s_{mb}-s_{nb})\cdot n_{1584}\right]/\left(n_{1584}\cdot n_{nb}\right)\right\}\cdot n_{nb}\\ s_{nc}=s_{mc}+\left\{\left[(s_{mc}-s_{nc})\cdot n_{1234}\right]/\left(n_{1234}\cdot n_{nc}\right)\right\}\cdot n_{nc} \end{array}$$

Based on above steps 1 to 4, the vectors of points located on the calculated motion trajectory lines were obtained. An angle vector **Λ_t_** = (*α_t_*, *β_t_*, *γ_t_*)^T^ was defined that describes the intersection angles between the motion trajectory lines defined in Section 4.1. A spatial line fitting process was performed on the measured vector sets of {**s_A,n_** = (*u_A,n_*, *v_A,n_*, *w_A,n_*)^T^|1 ≤ *n* ≤ *N*}, {**s_B,m_** = (*u_B,m_*, *v_B,m_*, *w_B,m_*)^T^|1 ≤ *m* ≤ *M*}, and {**s_C,k_** = (*u_C,k_*, *v_C,k_*, *w_C,k_*)^T^ |1 ≤ *k* ≤ *K* } and the unit vectors of fitted lines *l_u_*_2_, *l_v_*_2,_ and *l_w_*_2_, **n_u2_**, **n_v2_**, and **n_w2_**, were obtained, where **n_u2_** = (cosα_u2_, cosβ_u2_,cosγ_u2_)^T^, **n_v2_** = (cos*α_v_*_2_, cos*β_v_*_2_,cos*γ_v_*_2_)^T^, and **n_w1_** = (cos*α_w_*_2_, cos*β_w_*_2_,cos*γ_w_*_2_)^T^. The intersection angles, *α* (*l_u_*_2_ ∨ *l_v_*_2_), *β* (*l_u_*_2_ ∨ *l_w_*_2_), and *γ* (*l_w_*_2_ ∨ *l_v_*_2_) constitute an angle vector **Λ** = (*α*, *β*, *γ*)^T^, which were calculated by the dot product operation, i.e., *α* = arcos(**n_u2_**·**n_v2_**), *β* = arcos(**n_u2_**·**n_w2_**), and *γ* = arcos(**n_w2_**·**n_v2_**). The angle vector **Λ** represents the measured data of the motion trajectory lines and the angle vector **Λ_t_** represents its true value. The error vector between **Λ** and **Λ_t_** was calculated by the following equation:

(17) $$\Delta =(\delta_{\alpha },\delta_{\beta },\delta_{\gamma })=\Lambda -\Lambda_{t}$$

where **Δ** is the error vector. When a set of error indexes consisting of **s_p6_**, **Θ**, **n_a_**, **n_b_**, **n_c_**, **n_u1_**, **n_v1_**, and **n_w1_** was given, **Δ** was calculated by Equation (17) based on the above steps 1 to 4. **Δ** was used to analyze the influence of rotation angles of laser displacement sensor and gauge block on the orthogonality estimation of three-axis motion trajectories.

### 4.3. Simulation Results

The influence of the rotation angles on the accuracy of orthogonality estimation was simulated based on the following conditions: *L_S_* = 12 mm, *W_S_* = 12 mm, *H_S_* = 12 mm, **s_p6_** = (0,0,0)^T^, and *L*_0_ = 50. The simulation method limits the rotation angles, *θ_u_*, *θ_v_*, and *θ_w_*, within a small range and was set to be less than 10°. For the laser displacement sensor, *A*, the error was primarily from the variation of *α_a_*. Similarly, for sensors *B* and *C*, the error was primarily from the variation in *β_b_* and *γ_c_*, respectively. In the simulation, *l_u_*_1_, *l_v_*_1_, and *l_w_*_1_ were set to be closer to the U-, V-, and W-axis, respectively. *α_u_*_1_, *β_v_*_1_, and *γ_w_*_1_ were primarily considered in the simulation; their maximum values were set to be less than or equal to 10°. Based on the above analysis, the simulation was run under 10 categories of rotation angles.

The results obtained under the angles 1°–5° are listed in Table 2, which shows that when the rotation angle was less than 4°, the maximum absolute value of ***δ_α_***, ***δ_β_***, and***δ_γ_*** was 0.0043°, and the relative error compared with 90° was 0.005%. The results indicated that the effect of the rotation angles of the gauge block and the laser displacement sensors on the measurement results is completely negligible.

The results obtained under the angles 6°–10° are listed in Table 3. Table 2 and Table 3 shows that the minimum and maximum of absolute values of ***δ_α_***, ***δ_β_***, ***δ_γ_*** were 0.0103° and 0.0967°, respectively, and the relative error compared with 90° varied from 0.01% to0.09% when the rotation angles varied from 5° to 9°. When the rotation angles approached 10° the error was 0.1427° and the relative error compared with 90° was 0.16%.

We classified the error into three levels: low, middle, and large. Any rotation angle error less than 4° was considered small, the error under the rotation angles from 5° to 9° were classified as middle, and the error of rotation angles larger than 9° were considered large. Table 2 and Table 3 show that when the rotation angles of the laser displacement sensors and gauge block were less than 10°, the maximum error of motion trajectory orthogonality measurement was close to 0.09% which has little effect on the measurement.

## 5. Results and Discussion

**Figure 6:**
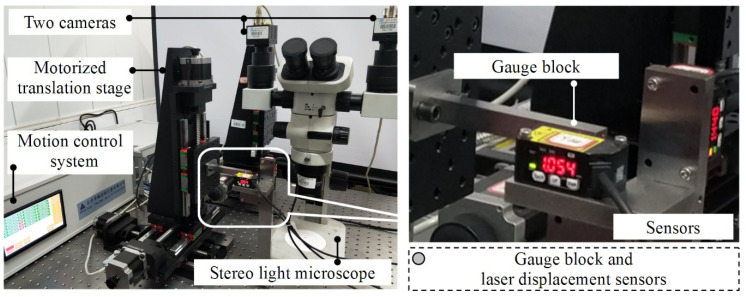
The photo of the experiment system. The system consists of an Olympus SZX7-style stereo light microscope, a 3-axis motorized translation stage with a positioning precision of 1μm, three Panasonic-style laser displacement sensors with a precision of 10μm and a gauge block with size of 90 mm(length) × 5 mm(width) × 5 mm(height) and surface roughness of 0.8 μm.

**Figure 7:**
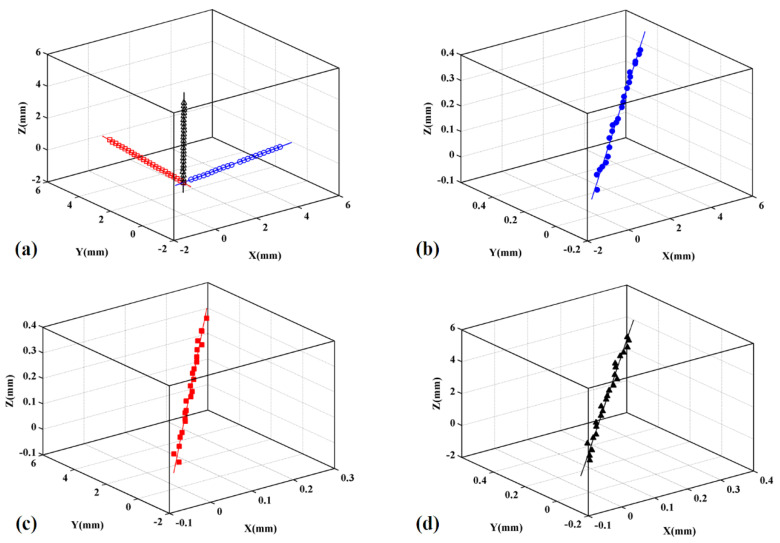
The fitted motion trajectory line in the X-, Y- and Z- directions. The three initial standard motion trajectory lines with 25 sampled points in each line were preset firstly which have the unit vectors along the X-, Y- and Z-axis, respectively. The angle between the two adjacent motion trajectory lines is initialized by 87.728 degrees. (**a**) The vectors of the sample points measured by the laser displacement sensors constructed three measured trajectory lines. (**b**) The fitted line is obtained based on the X-direction data; (**c**) The fitted line is obtained based on the Y-direction data; (**d**) The fitted line is obtained based on the Z-direction data.

The setup of the experimental system is shown in Figure 6. The system was placed on an optical platform, which consisted of a stereo light microscope, a three-axis motorized translation stage, a rectangular gauge block and three laser displacement sensors. The motion of the three-axis motorized translation stage was controlled in closed-loop mode. The measurement range of the laser displacement sensor was ±5 mm and its positioning precision was 0.01 mm. The gauge block was machined by precision machining and possessed a surface roughness of less than 0.0008 mm. The stage was rigorously assembled by the manufacturer. Virtual motion trajectories were designed for the experiments. The approach to generating the virtual motion trajectories is shown in Figure 7a. We assumed that the coordinate axes of O-XYZ were orthogonal, and three virtual motion trajectory lines that intersect with the X-, Y-, and Z-axes at small angles were created (generally less than 10°), separately. A series of sample points on the virtual motion trajectory lines was obtained and their vectors were regarded as true values. The gauge block moved along the virtual motion trajectory lines and passed sample points in order. When the gauge block reached a sample point, its position was measured by the laser displacement sensors. The measured positions of the sample points constituted the motion trajectories, and their spatial fitted lines were obtained, as shown in Figure 7b–d. The intersection angles of the three virtual motion trajectory lines were used as true values and the intersection angles of the three fitted lines were used as measured values. The error between the measured and true values was obtained. Six groups of experiments were performed to test the orthogonality of the motion trajectory lines. The range of the intersection angles as input parameters was set to [82.728°, 90°]. The results are listed in Table 4.

For the selection of the sample points on the motion trajectory line, we attempted to make the density of the sample points as large as possible. Here, we set 25 points for observation. For the spatial line fitting process, a gross error filter for gross error detection was used to obtain high-precision fitted lines, which needed several repeated fitting processes. If the vertical distance between the sample points and fitted line was larger than a given threshold (here, 0.02 mm was used), the sample points that exceeded the threshold were removed from the sample point sets. Then, the spatial line fitting was repeatedly performed on the remaining sample points until the satisfactory fitting precision was reached. In Table 4, *θ* is the intersection angle and includes three components, *θ_XY_*, *θ_XZ_*, and *θ_YZ_*, where *θ_XY_* is the angle between the X and *Y*-axis, *θ_XZ_* is the angle between the X- and *Z*-axis, and *θ_YZ_* is the angle between the Y- and *Z*-axis. In Table 4, the value of *θ* decreases gradually. When *θ* was 90°, 88.578°, 87.137°, 85.682°, 84.213° and 82.728°, the maximum absolute error was 0.125°, 0.161°, 0.206°, 0.245°, 0.146° and 0.160°, respectively, and the fluctuation range was 0.12°. The maximum relative errors of the six cases were 0.138%, 0.182%, 0.237%, 0.286%, 0.173% and 0.193%, respectively, and the fluctuation range was 0.148%. The results showed that the maximum absolute error of the measured value of *θ* was 0.245° and the maximum relative error was 0.286% when the value of *θ* changed from 90° to 82.728°.

**Figure 8:**
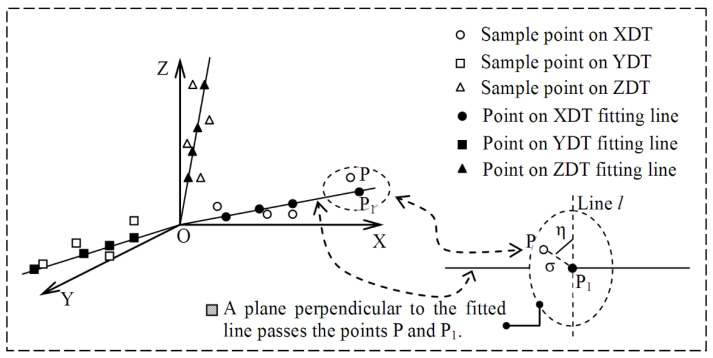
The definition of the error parameters of the fitted spatial line, where XDT = X-direction motion trajectory, YDT = Y-direction motion trajectory, ZDT = Z-direction motion trajectory. Two parameters, σ and η, are used. The parameter σ represents the distance between a point P and a fitted line, and point P_1_ is one of the fitted points corresponding to point P. The parameter η is used to describe the azimuth of a sample point, which is the intersection angle between the lines PP_1_ and *l*. The line *l* is located in the plane passing the points P_1_ and P, and perpendicular to the fitted line OP_1_.

**Figure 9:**
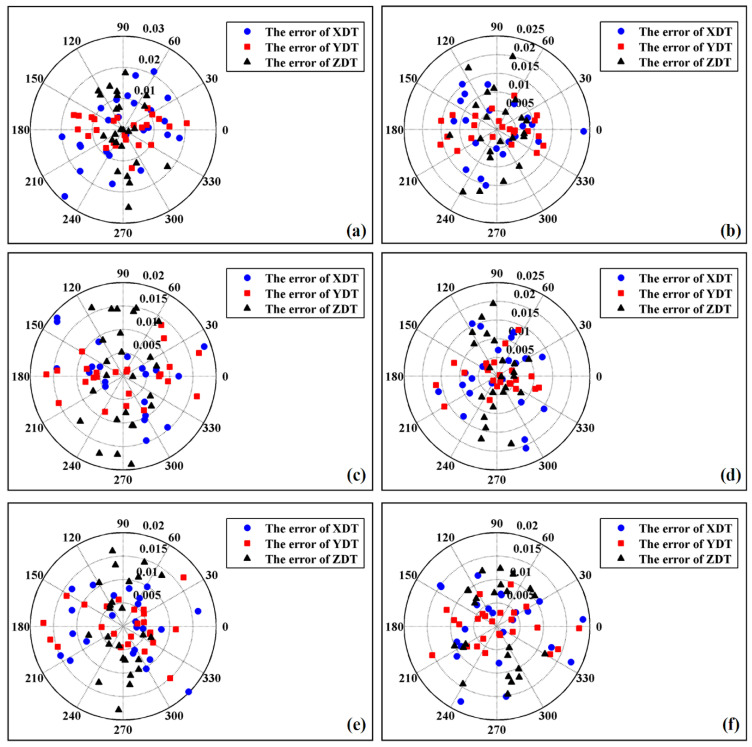
The error distribution of the fitted motion trajectory lines, where XDT = X-direction motion trajectory, YDT = Y-direction motion trajectory, ZDT = Z-direction motion trajectory. Six groups of experiments are designed to analyze the fitting errors under different angle parameters. The distribution of the errors is shown in (**a**–**f**). (**a**) The error was obtained under 90°; (**b**) The error was obtained under 88.578°; (**c**) The error was obtained under 87.137°; (**d**) The error was obtained under 85.683°; (**e**) The error was obtained under 84.213°; (**f**) The error was obtained under 82.728°.

The accuracy of spatial line fitting affected the precision of three-axis motion trajectory orthogonality measurement. Fitting errors in the six groups of experiments were counted and compared. The statistical rule of fitting error used in this paper is shown in Figure 8. Two types of parameters, σ and η, were defined to describe fitting error, where σ represents the vertical distance between the sample point and the fitted line, and η represents the azimuth of the sample point. The distribution of fitting error in polar coordinates is shown in Figure 9, where σ is the polar radius and η is the polar angle. The fitting error in Figure 9a–f was obtained under conditions 1–6 listed in Table 4. Figure 9a–f show that the distribution of sample points and their fitting error were random, and that the measured values of the sample points fluctuated. In Figure 9a–f, the values of *θ* are approximately 90°, 88.578°, 87.137°, 85.682°, 84.213° and 82.728°. Compared with the 90° motion trajectory lines, the motion trajectory lines generated under these six angles deviated by 0°, 1.422°, 2.863°, 4.318°, 5.787° and 7.272° respectively. From the results, the value of θ decreased gradually, and the degree of its corresponding motion trajectory lines deviating from the 90° motion trajectory line increased gradually. In Figure 9a–f, the number of sample points with fitting error more than 0.015 mm is 4, 1, 10, 1, 8 and 5 respectively, but the fitting error of all sample points is less than 0.02 mm. When the value of 90-*θ* was less than or equal to 7.272°, the maximum fitting error was 0.02 mm.

In Section 4.3, the influence of the rotation angles generated by the laser displacement sensor assembly on the orthogonality of the reference coordinate system was analyzed through simulation. If the influence is large, the accuracy of motion trajectory measurement is affected, and the angle deviation between the measured motion trajectory and the real motion trajectory is large. Figure 9a,b shows that the position offset between the sample points can be controlled within 0.02 mm, which indicated that the output data of the laser displacement sensor have good stability. In the experiment, the range of the deviation angles of the motion trajectory lines was 0°–7.272°. Table 4 shows that the absolute error of the deviation angles of the motion trajectory lines experienced small fluctuation: the maximum fluctuation was 0.245° and the corresponding relative error was 0.286%, which indicated that the measurement error of the motion trajectory lines was small. This experiment also showed that within this deviation angle range, the orthogonality of the reference coordinate system formed by laser displacement sensors has a weak influence on the angle measurement. This is consistent with the simulation results in Section 4.3.

## 6. Conclusions

Stereo light microscopes are designed based on a binocular stereo structure, which requires a large workspace and can be applied to develop microscopic stereovision systems and in the micromanipulation fields to provide micromanipulation robot systems with a stereovision function. The motion control system is an important part of micromanipulation robot systems. For a three-axis motion control system, the orthogonality and linearity of the three-axis motion trajectories have the accuracy of micromanipulation. So, the study of the mechanisms of the linearity and orthogonality of three-axis motion trajectories is important. Aiming at this problem, we constructed a novel approach to measure three-axis motion trajectories, designed the approximate orthogonal reference frame, and analyzed the influence of laser displacement sensor assembly error on the orthogonality of the reference frame via simulation. An approach to evaluate the linearity and orthogonality of three-axis motion trajectories was proposed, and then the rules to correct the non-orthogonal motion trajectories were developed. The results showed that when the assembly angles of the central-axis lines of the laser displacement sensors are limited within a range of 10°, the relative angle deviations of the axes of the reference frame compared with 90° are approximate to 0.09%, the accuracy of the axial motion trajectory fitting is approximately 0.02 mm, and the relative error of the intersection angles of the three-axis motion trajectories is approximately 0.3%. Thus, the results showed that when the assembly angle is limited within a reasonable range, the three-axis orthogonality of the orthogonal reference frame can be guaranteed, and the method proposed in this paper can be used to effective evaluate the orthogonality of three-axis motion trajectories for micromanipulation robot systems.

## Figures and Tables

**Table 1:** The orthogonal rules defined for motion trajectory lines.

Category	*α*	*β*	*γ*	Logic
Orthogonal rule	|*α*-90°| ≤ *T_abc_*	|*β*-90°| ≤ *T_abc_*	|*γ*-90°| ≤ *T_abc_*	*α&β&γ*
Non-orthogonal rule	|*α*-90°| > *T_abc_*	|*β*-90°| > *T_abc_*	|*γ*-90°| > *T_abc_*	*A||β||γ*

where *T_abc_* is a preset threshold for the angles *α*, *β* and *γ.*

**Table 2:** The effects of error sources on orthogonality estimation under the angles: 1°–5°.

Item	Parameter	Case I (1°)	Case II (2°)	Case III (3°)	Case IV (4°)	Case V (5°)
Input angle (°)	*θ_u_*, *θ_v_*, *θ_w_*	1.0, 1.0, 1.0	2.0, 2.0, 2.0	3.0, 3.0, 3.0	4.0, 4.0, 4.0	5.0, 5.0, 5.0
	*α_a_*, *β_a_*, *γ_a_*	0.9965, 89.2954, 89.2954	1.9925, 88.5912, 88.5912	2.9972, 87.8812, 87.8812	4.0000, 87.1727, 87.1727	4.9905, 86.4734, 86.4734
	*α_b_*, *β_b_*, *γ_b_*	89.2954, 0.9965, 89.2954	88.5912, 1.9925, 88.5912	87.8812, 2.9972, 87.8812	87.1727, 4.0000, 87.1727	86.4734, 4.9905, 86.4734
	*α_c_*, *β_c_*, *γ_c_*	89.2954, 89.2954, 0.9965	88.5912, 88.5912, 1.9925	87.8812, 87.8812, 2.9972	87.1727, 87.1727, 4.0000	86.4734, 86.4734, 4.9905
	*α_u_*_1_, *β_u_*_1_, *γ_u_*_1_	1.0006, 89.2925, 89.2925	2.0006, 88.5855, 88.5855	2.9994, 87.8796, 87.8796	4.0063, 87.1683, 87.1683	5.0008, 86.4662, 86.4662
	*α_v_*_1_, *β_v_*_1_, *γ_v_*_1_	89.2925, 1.0006, 89.2925	88.5855, 2.0006, 88.5855	87.8796, 2.9994, 87.8796	87.1683, 4.0063, 87.1683	86.4662, 5.0008, 86.4662
	*α_w_*_1_, *β_w_*_1_, *γ_w_*_1_	89.2925, 89.2925, 1.0006,	88.5855, 88.5855, 2.0006	87.8796, 87.8796, 2.9994	87.1683, 87.1683, 4.0063	86.4662, 86.4662, 5.0008
Output angle (°)	*α_u_*_2_, *β_u_*_2_, *γ_u_*_2_	1.7324, 90.2626, 88.2877	3.4640, 90.465, 86.5675	5.1940, 90.6086, 84.8419	6.9269, 90.6847, 83.1074	8.6494, 90.7093, 81.3801
	*α_v_*_2_, *β_v_*_2_, *γ_v_*_2_	88.3052, 1.7150, 90.2624	86.6374, 3.3945, 90.4638	84.9989, 5.0376, 90.6034	83.3849, 6.6496, 90.6729	81.8102, 8.2190, 90.6869
	*α_w_*_2_, *β_w_*_2_, *γ_w_*_2_	90.2802, 88.3050, 1.7181	90.5365, 86.6359, 3.4067	90.7698, 84.9940, 5.0651	90.9738, 83.3742, 6.6976	91.1644, 81.7907, 8.2926
	*α_t_*, *β_t_*, *γ_t_*	88.5763, 88.5763, 88.5763	87.1369, 87.1369, 87.1369	85.6835, 85.6835, 85.6835	84.2030, 84.2030, 84.2030	82.7265, 82.7265, 82.7265
	*α*, *β*, *γ*	88.5763, 88.5764, 88.5763	87.1367, 87.1372, 87.1370	85.6821, 85.6847, 85.6838	84.1987, 84.2067, 84.2039	82.7162, 82.7353, 82.7289
	*δ_α_*, *δ_β_*, *δ_γ_*	0, 0.0001, 0	−0.0002, 0.0003, 0.0001	−0.0014, 0.0012, 0.0003	−0.0043, 0.0037, 0.0009	−0.0103, 0.0088, 0.0024
Influence level		Weak (<0.005°)	Weak (<0.005°)	Weak (<0.005°)	Weak (<0.005°)	Middle (0.01° <&<0.1°)

**Table 3:** The effects of error sources on orthogonality estimation under the angles: 6°–10°.

Item	Parameter	Case VI (6°)	Case VII (7°)	Case VIII (8°)	Case IX (9°)	Case X (10°)
Input angle (°)	*θ_u_*, *θ_v_*, *θ_w_*	6.0, 6.0, 6.0	7.0, 7.0, 7.0	8.0, 8.0, 8.0	9.0, 9.0, 9.0	10.0, 10.0, 10.0
	*α_a_*, *β_a_*, *γ_a_*	5.9978, 85.7628, 85.7628	7.0013, 85.0555, 85.0555	8.0005, 84.3520, 84.3520	8.9997, 83.6494, 83.6494	10.0002, 82.9468, 82.9468
	*α_b_*, *β_b_*, *γ_b_*	85.7628, 5.9978, 85.7628	85.0555, 7.0013, 85.0555	84.3520, 8.0005, 84.3520	83.6494, 8.9997, 83.6494	82.9468, 10.0002, 82.9468
	*α_c_*, *β_c_*, *γ_c_*	85.7628, 85.7628, 5.9978	85.0555, 85.0555, 7.0013	84.3520, 84.3520, 8.0005	83.6494, 83.6494, 8.9997	82.9468, 82.9468, 0.0002
	*α_u_*_1_, *β_u_*_1_, *γ_u_*_1_	6.0022, 85.7597, 85.7597	6.9900, 85.0634, 85.0634	8.0032, 84.3501, 84.3501	9.0016, 83.6481, 83.6481	10.0003, 82.9468, 82.9468
	*α_v_*_1_, *β_v_*_1_, *γ_v_*_1_	85.7597, 6.0022, 85.7597	85.0634, 6.9900, 85.0634	84.3501, 8.0032, 84.3501	83.6481, 9.0016, 83.6481	82.9468, 10.0003, 82.9468
	*α_w_*_1_, *β_w_*_1_, *γ_w_*_1_	85.7597, 85.7597, 6.0022,	85.0634, 85.0634, 6.9900,	84.3501, 84.3501, 8.0032	83.6481, 83.6481, 9.0016	82.9468, 82.9468, 10.0003
Output angle (°)	*α_u_*_2_, *β_u_*_2_, *γ_u_*_2_	10.3714, 90.6677, 79.6506	12.0792, 90.5764, 77.9350	13.7932, 90.4053, 76.2130	15.4882, 90.1872, 74.5130	17.1705, 89.9113, 72.8298
	*α_v_*_2_, *β_v_*_2_, *γ_v_*_2_	80.2634, 9.7574, 90.6302	78.7582, 11.2541, 90.5186	77.2719, 12.7323, 90.3209	75.8295, 14.1707, 90.0696	74.4226, 15.5794, 89.7532
	*α_w_*_2_, *β_w_*_2_, *γ_w_*_2_	91.3280, 80.2321, 9.859	91.4811, 78.7122, 11.3871	91.5940, 77.2081, 12.8942	91.6993, 75.7455, 14.3597	91.7859, 74.3162, 15.7903
	*α_t_*, *β_t_*, *γ_t_*	81.2261, 81.2261, 81.2261	79.7333, 79.7333, 79.7333	78.1896, 78.1896, 78.1896	76.6567, 76.6567, 76.6567	75.1121, 75.1121, 75.1121
	*α*, *β*, *γ*	81.2053, 81.2443, 81.2313	79.6957, 79.7664, 79.7431	78.1274, 78.2453, 78.2070	76.5600, 76.7443, 76.6853	74.9694, 75.2433, 75.1569
	*δ_α_*, *δ_β_*, *δ_γ_*	−0.0208, 0.0182, 0.0052	−0.0376, 0.0331, 0.0098	−0.0622, 0.0557, 0.0174	−0.0967, 0.0876, 0.0286	−0.1427, 0.1312, 0.0448
Influence level		Middle (0.01° < & < 0.1°)	Middle (0.01° < & < 0.1°)	Middle (0.01° < & < 0.1°)	Middle (0.01° < & < 0.1°)	Large (>0.1°)

**Table 4:** The results of angle measurement between moving trajectories.

Group	*θ*	True Value (°)	Measured Value (°)	Absolute Error (°)	Relative Error (%)
1	*θ_XY_*	90.000	89.875	−0.125	0.138
	*θ_XZ_*	90.000	90.015	0.015	0.016
	*θ_YZ_*	90.000	89.943	−0.057	0.063
2	*θ_XY_*	88.578	88.467	−0.111	0.125
	*θ_XZ_*	88.578	88.417	−0.161	0.182
	*θ_YZ_*	88.578	88.663	0.085	0.096
3	*θ_XY_*	87.137	86.931	−0.206	0.237
	*θ_XZ_*	87.137	87.070	−0.067	0.077
	*θ_YZ_*	87.137	87.106	−0.031	0.035
4	*θ_XY_*	85.682	85.720	0.038	0.045
	*θ_XZ_*	85.682	85.437	−0.245	0.286
	*θ_YZ_*	85.682	85.597	−0.085	0.099
5	*θ_XY_*	84.213	84.178	−0.035	0.042
	*θ_XZ_*	84.213	84.067	−0.146	0.173
	*θ_YZ_*	84.213	84.203	−0.010	0.012
6	*θ_XY_*	82.728	82.623	−0.105	0.127
	*θ_XZ_*	82.728	82.568	−0.160	0.193
	*θ_YZ_*	82.728	82.809	0.081	0.098
